# Transanal total mesorectal excision combined with intersphincteric resection has similar long-term oncological outcomes to laparoscopic abdominoperineal resection in low rectal cancer: a propensity score-matched cohort study

**DOI:** 10.1093/gastro/goac026

**Published:** 2022-06-14

**Authors:** Zhi-Hang Liu, Zi-Wei Zeng, Hai-Qing Jie, Liang Huang, Shuang-Ling Luo, Wen-Feng Liang, Xing-Wei Zhang, Liang Kang

**Affiliations:** Department of Colorectal Surgery, The Sixth Affiliated Hospital, Sun Yat-sen University, Guangzhou, Guangdong, P. R. China; Guangdong Provincial Key Laboratory of Colorectal and Pelvic Floor Diseases, The Sixth Affiliated Hospital, Sun Yat-sen University, Guangzhou, Guangdong, P. R. China; Department of Colorectal Surgery, The Sixth Affiliated Hospital, Sun Yat-sen University, Guangzhou, Guangdong, P. R. China; Guangdong Provincial Key Laboratory of Colorectal and Pelvic Floor Diseases, The Sixth Affiliated Hospital, Sun Yat-sen University, Guangzhou, Guangdong, P. R. China; Department of Colorectal Surgery, The Sixth Affiliated Hospital, Sun Yat-sen University, Guangzhou, Guangdong, P. R. China; Guangdong Provincial Key Laboratory of Colorectal and Pelvic Floor Diseases, The Sixth Affiliated Hospital, Sun Yat-sen University, Guangzhou, Guangdong, P. R. China; Department of Colorectal Surgery, The Sixth Affiliated Hospital, Sun Yat-sen University, Guangzhou, Guangdong, P. R. China; Guangdong Provincial Key Laboratory of Colorectal and Pelvic Floor Diseases, The Sixth Affiliated Hospital, Sun Yat-sen University, Guangzhou, Guangdong, P. R. China; Department of Colorectal Surgery, The Sixth Affiliated Hospital, Sun Yat-sen University, Guangzhou, Guangdong, P. R. China; Guangdong Provincial Key Laboratory of Colorectal and Pelvic Floor Diseases, The Sixth Affiliated Hospital, Sun Yat-sen University, Guangzhou, Guangdong, P. R. China; Department of Colorectal Surgery, The Sixth Affiliated Hospital, Sun Yat-sen University, Guangzhou, Guangdong, P. R. China; Guangdong Provincial Key Laboratory of Colorectal and Pelvic Floor Diseases, The Sixth Affiliated Hospital, Sun Yat-sen University, Guangzhou, Guangdong, P. R. China; Department of Colorectal Surgery, The Sixth Affiliated Hospital, Sun Yat-sen University, Guangzhou, Guangdong, P. R. China; Guangdong Provincial Key Laboratory of Colorectal and Pelvic Floor Diseases, The Sixth Affiliated Hospital, Sun Yat-sen University, Guangzhou, Guangdong, P. R. China; Department of Colorectal Surgery, The Sixth Affiliated Hospital, Sun Yat-sen University, Guangzhou, Guangdong, P. R. China; Guangdong Provincial Key Laboratory of Colorectal and Pelvic Floor Diseases, The Sixth Affiliated Hospital, Sun Yat-sen University, Guangzhou, Guangdong, P. R. China

**Keywords:** rectal cancer, transanal total mesorectal excision, laparoscopic abdominoperineal resection, oncological outcomes

## Abstract

**Background:**

Transanal total mesorectal excision (taTME) or intersphincteric resection (ISR) has recently proven to be a valid and safe surgical procedure for low rectal cancer. However, studies focusing on the combination of these two technologies are limited. This study aimed to evaluate perioperative results, long-term oncologic outcomes, and anorectal functions of patients with low rectal cancer undergoing taTME combined with ISR, by comparing with those of patients undergoing laparoscopic abdominoperineal resection (laAPR).

**Methods:**

After 1:1 propensity score matching, 200 patients with low rectal cancer who underwent laAPR (*n *=* *100) or taTME combined with ISR (*n *=* *100) between September 2013 and November 2019 were included. Patient demographics, clinicopathological characteristics, oncological outcomes, and anal functional results were analysed.

**Results:**

Patients in the taTME-combined-with-ISR group had less intraoperative blood loss (79.6 ± 72.6 vs 107.3 ± 65.1 mL, *P *=* *0.005) and a lower rate of post-operative complications (22.0% vs 44.0%, *P *<* *0.001) than those in the laAPR group. The overall local recurrence rates were 7.0% in both groups within 3 years after surgery. The 3-year disease-free survival rates were 86.3% in the taTME-combined-with-ISR group and 75.1% in the laAPR group (*P *=* *0.056), while the 3-year overall survival rates were 96.7% and 94.2%, respectively (*P *=* *0.319). There were 39 patients (45.3%) in the taTME-combined-with-ISR group who developed major low anterior resection syndrome, whereas 61 patients (70.9%) had good post-operative anal function (Wexner incontinence score ≤ 10).

**Conclusion:**

We found similar long-term oncological outcomes for patients with low rectal cancer undergoing laAPR and those undergoing taTME combined with ISR. Patients receiving taTME combined with ISR had acceptable post-operative anorectal function.

## Introduction

Total mesorectal excision (TME) is standard treatment for patients with middle or low rectal cancer, which means to prevent local recurrence and improve overall survival (OS) rates [1, 2]. Abdominoperineal resection (APR) is classic surgery for low rectal cancer patients. Although the laparoscopic technique is used to reduce the abdominal wound, APR still involves a resection of the anus, resulting in a huge wound [3–5]. After receiving APR, patients will suffer additional physical and psychological burden due to the loss of anal sphincter function [6, 7]. Therefore, intersphincteric resection (ISR) is considered an anus-preserving treatment of low rectal cancer [8], which is expected to be a safe alternative to APR based on similar oncological outcomes under some conditions [9].

Compared with traditional open surgery, the use of laparoscopy in rectal cancer can reduce post-operative hospital stay and surgical wound complications. Moreover, large randomized–controlled trials such as the American College of Surgeons Oncology Group (ACOSOG) trial and Australasian Laparoscopic Cancer of the Rectum Trial (AlaCaRT) have suggested that laparoscopic surgery is not inferior to open surgery in rectal cancer oncological outcomes [10, 11]. However, open or laparoscopic surgery is still a transabdominal approach. With limited space for moving the distal rectum, surgeons cannot accurately determine the location of the tumor no matter which top–down surgery (APR, ISR, or others) they choose for low rectal cancer. This affects the accurate separation of tumors by surgeons. When patients have narrow pelvis or high body mass index (BMI), this situation will be worse. The transanal total mesorectal excision (taTME) process is known as a “bottom-to-up” approach to obtain clear surgical planes, which may reduce the difficulty of excision and improve the quality of surgical specimens [12]. A number of studies have focused on comparing the short-term and long-term outcomes of laparoscopic TME (laTME) and taTME, and the results were similar [13–16].

Transanal endoscopic technology has a high-definition magnification effect and distal rectal expansion effect, which can help surgeons accurately locate and remove the tumors. At the same time, ISR can protect the patient’s external sphincter and even part of the internal sphincter to ensure the patient’s autonomous defecation function without compromising oncological outcomes. Although ISR and taTME are known as surgical options for low rectal cancer, studies focusing on the combination of these two technologies are limited [17].

To address this knowledge gap, we conducted this study to compare laparoscopic abdominoperineal resection (laAPR) and taTME combined with ISR regarding the results of operation, pathology, oncological outcomes, and anorectal function in low rectal cancer. Based on the characteristics of the two surgical approaches, we assumed that taTME combined with ISR could improve operative results and have satisfactory oncological and functional outcomes.

## Patients and methods

### Study design

Patients with rectal cancer from the Sixth Affiliated Hospital of Sun Yat-sen University (Guangzhou, China) who underwent laAPR or taTME combined with ISR between September 2013 and November 2019 were included in this study. The data of all patients were collected from the colorectal cancer database.

Case inclusion criteria were as follows: (i) biopsy-proven rectal adenocarcinoma; (ii) the height of each tumor from the anal verge was ≤5 cm based on magnetic resonance imaging (MRI) reports; (iii) T1–3N0–2 lesions without threatening mesorectal fascia by MRI and computed tomography (CT) reports; and (iv) undergoing radical resection of rectal cancer. Exclusion criteria were as follows: (i) TME could not be performed; (ii) emergency surgery with intestinal obstruction or perforation; (iii) a history of colorectal surgery; (iv) distant metastasis was found in patients with rectal cancer before surgery by CT report or positron emission tomography (PET)-CT report; (v) the occurrence of other systemic tumors such as liver cancer or lung cancer; or (vi) fecal incontinence or constipation.

In order to reduce the selection bias in our observational study, we matched the two groups of patients with a ratio of 1:1 according to the following 10 covariates: gender, age, BMI, American Society of Anesthesiologists category (ASA), neoadjuvant radiotherapy, tumor distance between the tumor and the anus verge, tumor size, clinical T category, N category, and TNM-stage [18]. The following data were all collected from the colorectal cancer database of our hospital: preoperative clinical data including gender, age, and BMI; the distance between tumor and the anal verge; tumor size; the ratio of neoadjuvant radiotherapy; preoperative tumor stage; intraoperative data including operation time and blood loss; post-operative hospital stay; intraoperative and post-operative complications; the Clavien–Dindo classification; and pathological results including mesorectal resection quality, the rate of positive distal resection margin, the status of circumferential resection margin, differentiation grade, and post-operative tumor stage.

## Chemoradiotherapy

Patients who had clinical Stage I tumors or contraindications of neoadjuvant chemoradiotherapy were recommended to receive surgery directly. Patients having a T3 lesion with or without N+ were recommended to undergo neoadjuvant chemoradiotherapy, which was based on fluorouracil-type drugs.

Capecitabine alone, fluorouracil, or fluorouracil + leucovorin were recommended for neoadjuvant chemotherapy. Neoadjuvant chemotherapy was administered simultaneously during neoadjuvant radiotherapy. There were two neoadjuvant radiotherapy schemes based on different patient conditions. The first scheme was a short-duration neoadjuvant radiotherapy scheme, 5 Gy each time, five times, and 25 Gy in 1 week. The other scheme was a long neoadjuvant radiotherapy scheme, 1.8–2.0 Gy each time, 25–28 times, and 45.0–50.4 Gy in total, which was more commonly used. The patients received neoadjuvant therapy and had a rest for 8 weeks before surgery.

After receiving radical resection, patients with Stage II–III rectal cancer were recommended to receive chemoradiotherapy first and then received adjuvant chemotherapy. The other option was the sandwich treatment mode. After undergoing one or two cycles of adjuvant chemotherapy, patients received chemoradiotherapy and finally received adjuvant chemotherapy.

### Surgery treatment

In low rectal cancer, T3 means that tumor invades the intersphincteric groove, but does not invade the external sphincter. The surgery involved in this study was only performed in patients with clinical T1–3 tumors that had a negative circumferential resection margin. There was no evidence of tumor involvement with the external anal sphincter or levator ani muscles.

All patients were operated on by experienced surgeons (who had each performed >200 rectal operations per year) of our hospital. In the laAPR procedure, after laparoscopic TME, the patients’ anus and perianal tissue were removed; all patients received conventional sigmoid end colostomy. In the taTME-combined-with-ISR procedure, surgeons performed purse strings suture at the lower margin of the tumor ∼1–2 cm to isolate the tumor. When the tumor height was >3 cm from the anal verge, surgeons would place the port in the anus, separate and retain part of the internal sphincter, separate the internal and external sphincter with transanal endoscopy, and then perform the subsequent taTME. Such partial ISR was performed in 59 patients. When the tumor height was ≤3 cm from the anal verge, surgeons would perform separation of the internal and external sphincter, then place the port in the anus and perform subsequent taTME with transanal endoscopy. Complete ISR was performed in 41 patients. We finally used an end-to-end anastomosis for reconstruction. When the height of anastomosis was 1–2 cm from the anal verge, hand-sewn anastomosis was performed by surgeons. When this height increased, surgeons tended to perform stapled anastomosis. Other details of the taTME procedure have been published previously [19].

### Follow-up schedule

After surgery, patients were scheduled to undergo physical examination and measurement of carcinoembryonic antigen and CA19-9 every 3 months for 2 years, every 6 months for 3 years, and then every year after 5 years. Patients underwent chest, abdomen, and pelvic CT or MRI every 6 months for 2 years, and yearly thereafter up to 5 years post-operatively. Endoscopy was scheduled to be performed within 1 year after the operation. If there was any abnormality, the patients were suggested to be re-examined within 1 year; if no polyps were found, they were suggested to be re-examined within 3 years, and then once every 5 years. Colorectal adenomas that appeared during follow-up examinations were recommended to be resected. The PET-CT examination was not a routinely recommended examination item. For patients with existing or suspected recurrence and distant metastasis, PET-CT examination could be considered to exclude recurrence and metastasis.

## End point

The primary end points included the 3-year local recurrence (LR) rates, 3-year disease-free survival (DFS) rates, and 3-year OS rates. Secondary end points were the early post-operative results, histopathological results, and anorectal function.

### Functional assessment

Patient anorectal function was assessed by direct telephone interviews. The questionnaires followed the validated low anterior resection syndrome (LARS) score scale [20] and Wexner incontinence score scale [21].

The LARS score scale is a simple tool for quick evaluation of anorectal function after a rectal cancer operation. The scoring system includes incontinence for flatus and liquid stools, frequency of bowel movements, stool clustering, and urgency. There are three grades based on the total score: no LARS (0–20), minor LARS (21–29), and major LARS (30–42).

The level of severity of fecal incontinence is measured by five questions including incontinence frequency of solid, liquid, and gas; the frequency of wearing pads; and lifestyle alteration. Each question has five levels (0, never; 1, rarely; 2, sometimes; 3, usually; 4, always) with scores adding up to acquire the Wexner continence score (0 means perfect continence, whereas 20 means complete incontinence). Anal function is considered “good” if the Wexner score is ≤10 and “poor” when it is >10 [22].

### Statistical analysis

Data were analysed by the Statistical Package for Social Science (SPSS 25.0; SPSS Inc., Chicago, IL, USA) and are presented as number of patients, percentage, mean ± standard deviation (SD) (for normally distributed data), and median and range (for non-normally distributed data). Differences between the two groups were assessed using a *t*-test, Pearson Chi-square (*χ*^2^) test, Fisher exact test, or Mann–Whitney *U* test as appropriate. A *P*-value of <0.05 was considered statistically significant. Under 0.1 caliper distance, propensity score matching (PSM) was used with a 1:1 nearest neighbor matching algorithm and unmatched patients were excluded.

## Results

### Patient characteristics

**Figure 1.**
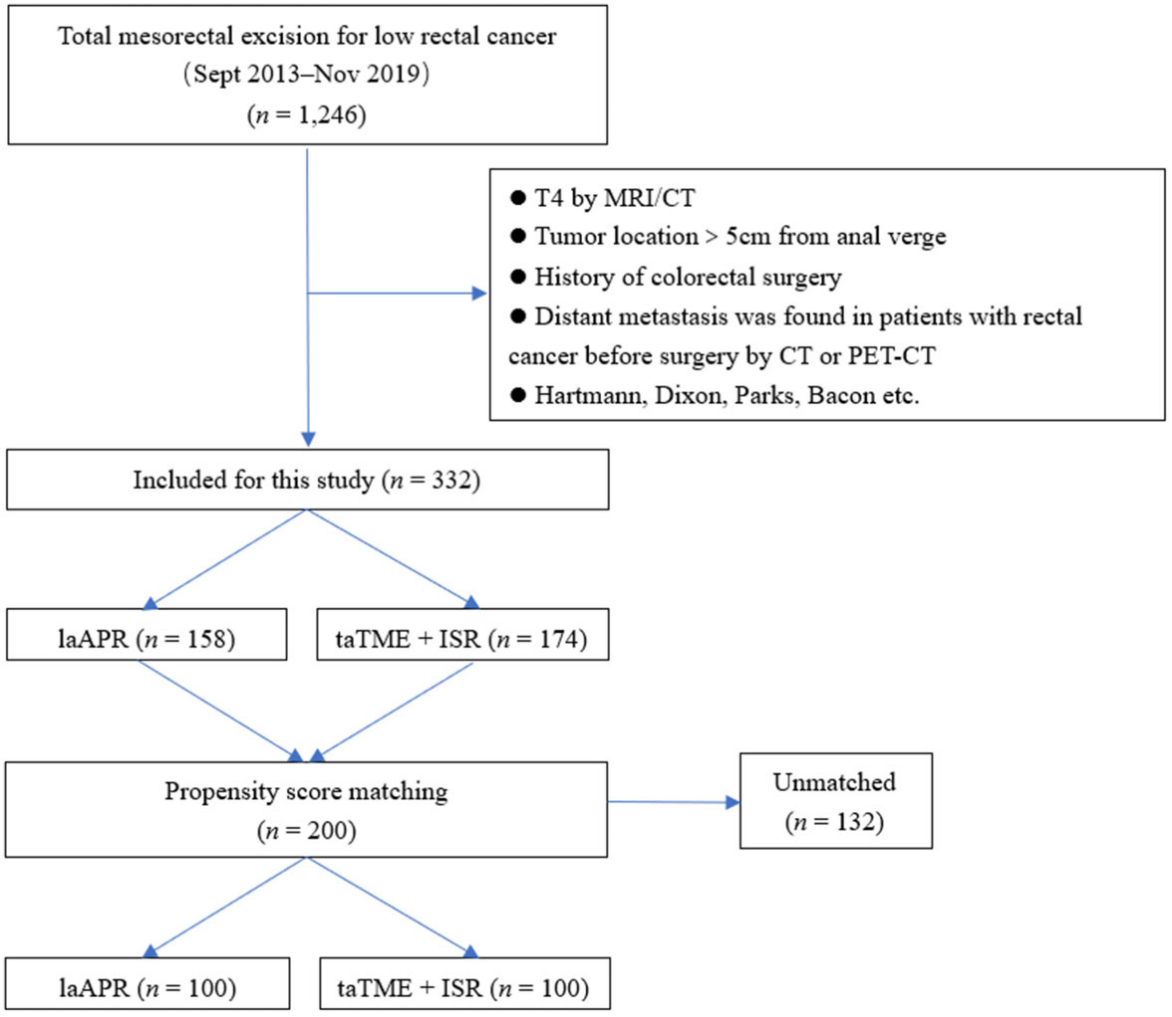
Patient selection diagram. laAPR, laparoscopic abdominoperineal resection; taTME, transanal total mesorectal excision; ISR, intersphincteric resection.

At the beginning, 1,246 patients who underwent TME for low rectal cancer were included in this study. Before matching, 158 patients in the laAPR group and 174 patients in the taTME-combined-with-ISR group met the inclusion criteria and had significant difference in age, neoadjuvant therapy, tumor distance from the anus verge, preoperative T category, and preoperative clinical stage. After a 1:1 PSM process, 200 patients (the laAPR group, *n *=* *100; the taTME-combined-with-ISR group, *n *=* *100) were included. The patients underwent surgery between September 2013 and November 2019. The details of the PSM process are shown in Figure 1 and baseline characteristics are shown in Table 1. No significant difference was observed regarding all preoperative data. The proportions of male patients were higher than those of female patients in both groups. However, this proportion was not significantly different between the two groups (69% in the laAPR group vs 65% in the taTME-combined-with-ISR group, *P *=* *0.652). Although more patients were treated with neoadjuvant therapy in the taTME-combined-with-ISR group than in the laAPR group, no significance was reached (47.0% vs 36.0%, *P *=* *0.151). The average distance between the tumor and the anus was 3.2 cm (range, 1.1–5.0 cm) in the laAPR group and 3.3 cm (range, 1.7–5.0 cm) in the taTME-combined-with-ISR group (*P *=* *0.189). Similarly, there was no statistical difference between these two groups in terms of tumor size, preoperative T category, preoperative N category, and preoperative stage.

**Table 1.** Demographic and clinical characteristics of patients

	Unmatched patients			Matched patients		
Characteristic	laAPR	taTME + ISR	*P*-value	laAPR	taTME + ISR	*P*-value
	(*n *=* *158)	(*n *=* *174)		(*n *=* *100)	(*n *=* *100)	
Gender, *n* (%)			0.484			0.652
Male	103 (65.2)	120 (69.0)		69 (69.0)	65 (65.0)	
Female	55 (34.8)	54 (31.0)		31 (31.0)	35 (35.0)	
Age, years, mean ± SD	59.9 ± 12.9	56.9 ± 12.4	0.032	59.1 ± 12.7	56.97 ± 10.5	0.207
BMI, mean ± SD	22.1 ± 3.1	22.6 ± 3.0	0.115	22.3 ± 3.2	22.5 ± 2.9	0.623
ASA classification, *n* (%)			0.486			0.506
I	115 (72.8)	133 (76.4)		68 (68.0)	63 (63.0)	
II	39 (24.7)	36 (20.7)		29 (29.0)	34 (34.0)	
III	4 (2.5)	5 (2.9)		3 (3.0)	3 (3.0)	
Neoadjuvant therapy, *n* (%)			<0.001			0.151
Yes	48 (30.4)	87 (50.0)		36 (36.0)	47 (47.0)	
No	110 (69.6)	87 (50.0)		64 (64.0)	53 (53.0)	
Neoadjuvant radiotherapy, *n* (%)			0.124			0.216
Yes	24 (15.2)	38 (21.8)		16 (16.0)	24 (24.0)	
No	134 (84.8)	136 (78.2)		84 (84.0)	76 (76.0)	
Tumor distance from the anal verge, cm, mean ± SD	2.9 ± 0.9	3.7 ± 0.9	<0.001	3.2 ± 0.9	3.3 ± 0.8	0.189
Tumor size, cm, mean ± SD	3.3 ± 1.2	3.2 ± 1.2	0.361	3.1 ± 1.1	3.2 ± 1.1	0.523
Preoperative T category, *n* (%)			0.002			0.455
T1	4 (2.5)	4 (2.3)		3 (3.0)	3 (3.0)	
T2	18 (11.4)	46 (26.4)		17 (17.0)	22 (22.0)	
T3	136 (86.1)	124 (71.3)		80 (80.0)	75 (75.0)	
Preoperative N category, *n* (%)			0.043			0.671
N0	86 (54.4)	112 (64.4)		70 (70.0)	67 (67.0)	
N1	51 (32.3)	49 (28.2)		25 (25.0)	27 (27.0)	
N2	21 (13.3)	13 (7.5)		5 (5.0)	6 (6.0)	
Preoperative clinical stage, *n* (%)			0.016			0.895
I	20 (12.7)	38 (21.8)		19 (19.0)	21 (21.0)	
II	65 (41.1)	74 (42.5)		51 (51.0)	46 (46.0)	
III	73 (46.2)	62 (35.6)		30 (30.0)	33 (33.0)	

laAPR, laparoscopic abdominoperineal resection; taTME, transanal total mesorectal excision; ISR, intersphincteric resection; SD, standard deviation; BMI, body mass index; ASA, American Society of Anesthesiologists.

### Perioperative outcomes

Perioperative results are presented in Table 2. The mean operative time in the laAPR group was significantly longer than that in the taTME-combined-with-ISR group (262.4 ± 81.2 vs 222.1 ± 76.1 min, *P *<* *0.001). The average intraoperative blood loss was higher in the laAPR group than in the taTME group (107.3 ± 65.1 vs 79.6 ± 72.6 mL, *P *=* *0.005). All patients in the laAPR group received conventional sigmoid colostomy, whereas 65 patients in the taTME group underwent preventive ostomy (*P *<* *0.001). No patients in the two groups had intraoperative complications. The rate of post-operative complications in the laAPR group was higher than that in the taTME-combined-with-ISR group (44.0% vs 22.0%, *P *=* *0.001). Post-operative hospital stay in the laAPR group was significantly longer than that in the taTME-combined-with-ISR group (19.5 ± 10.0 vs 10.0 ± 4.6 days, *P *<* *0.001). Some patients in the laAPR group developed incision infection (15.0%), fat liquefaction (12.0%), and stoma necrosis (4.0%), whereas the patients in the taTME-combined-with-ISR group did not. Certain patients in the taTME-combined-with-ISR group experienced anastomotic leakage (11.0%), anastomotic stenosis (2.0%), and rectovaginal fistula (3.0%), whereas the patients in the taTME-combined-with-ISR group did not. Though more patients received a secondary operation due to anastomotic leakage in the taTME-combined-with-ISR group (5.0%) than in the laAPR group (3.0%), they showed no significant difference (*P *=* *0.721). None of the patients died within 30 days following surgery in both groups.

**Table 2.** Perioperative outcomes

Factor	laAPR (*n *=* *100)	taTME + ISR (*n *=* *100)	*P-*value
Operative time in minutes, mean ± SD (range)	262.4 ± 81.2	222.1 ± 76.1	<0.001
Intraoperative blood loss in ml, mean ± SD (range)	107.3 ± 65.1	79.6 ± 72.6	0.005
Conversion, *n* (%)	0	0	1
Stoma, *n* (%)	100 (100.0)	65 (65.0)	<0.001
Post-operative hospital stays in days, mean ± SD	19.5 ± 10.0	10.0 ± 4.6	<0.001
Post-operative complications, *n* (%)	44 (44.0)	22 (22.0)	0.001
Anastomotic leakage	0	11 (11.0)	
Anastomotic stenosis	0	2 (2.0)	
Intestinal obstruction	8 (8.0)	5 (5.0)	
Presacral abscess	1 (1.0)	0	
Incision infection	15 (15.0)	0	
Pulmonary infection	2 (2.0)	0	
Fat liquefaction	12 (12.0)	0	
Urinary retention	1 (1.0)	1 (1.0)	
Stoma necrosis	4 (4.0)	0	
Parastomal hernia	1 (1.0)	0	
Rectovaginal fistula	0	3 (3.0)	
Clavien–Dindo classification, *n* (%)			0.003
None	56 (56.0)	78 (78.0)	
I	9 (9.0)	1 (1.0)	
II	29 (29.0)	16 (16.0)	
III	6 (6.0)	5 (5.0)	
Secondary operation due to complications, *n* (%)	3 (3.0)	5 (5.0)	0.721
Death within 30 days, *n* (%)	0	0	1

laAPR, laparoscopic abdominoperineal resection; taTME, transanal total mesorectal excision; ISR, intersphincteric resection; SD, standard deviation.

### Pathological results

Pathological results are demonstrated in Table 3. No significant difference between the two groups was observed in the quality of mesorectal specimens (*P *=* *0.748). The length of resected intestine and the distance between the tumor and the distal resection margin (DRM) for the laAPR group were longer than those for the taTME-combined-with-ISR group (16.9 ± 8.3 vs 12.3 ± 7.8 cm, *P *<* *0.001; 3.0 ± 1.2 vs 0.9 ± 0.8 cm, *P *<* *0.001). With regard to specimen margins, there was no positive DRM result in either group; the circumferential margin (CRM) was positive in one patient in the taTME-combined-with-ISR group vs none in the laAPR group. In addition, the proportion of pathological stages between the two groups did not differ significantly (*P *=* *0.854). As depicted in pathological results, the majority of patients had T3 diseases (80% in the laAPR group vs 75% in the taTME-combined-with-ISR group).

**Table 3.** Post-operative histopathological data

Factor	laAPR (*n *=* *100)	taTME + ISR (*n *=* *100)	*P*-value
Length of resected intestine, cm, means ± SD (range)	16.9 ± 8.3	12.3 ± 7.8	<0.001
Quality of mesorectal resection specimen, *n* (%)			0.748
Complete	94 (94.0)	96 (96.0)	
Nearly complete	6 (6.0)	4 (4.0)	
Incomplete	0	0	
Length between tumor and distal resection margin, cm, mean ± SD	3.0 ± 1.2	0.9 ± 0.8	<0.001
Positive distal resection margin, *n* (%)	0	0	–
Positive circumferential margin, *n* (%)	0	1 (1.0)	1
Lymph node harvest, *n* (%)	14.6 ± 7.1	15.0 ± 7.9	0.661
Differentiation status, *n* (%)			0.863
pCR	10 (10.0)	8 (8.0)	
Low-grade	8 (8.0)	3 (3.0)	
Moderate-grade	66 (66.0)	81 (81.0)	
High-grade	16 (16.0)	8 (8.0)	
Post-operative T category, *n* (%)			0.966
pCR	10 (10.0)	8 (8.0)	
T1	3 (3.0)	7 (7.0)	
T2	36 (36.0)	33 (33.0)	
T3	51 (51.0)	52 (52.0)	
Post-operative N category, *n* (%)			0.477
N0	75 (75.0)	79 (79.0)	
N1	18 (18.0)	17 (17.0)	
N2	7 (7.0)	4 (4.0)	
Post-operative pathological stage, *n* (%)			0.725
pCR	10 (10.0)	8 (8.0)	
I	32 (32.0)	36 (36.0)	
II	33 (33.0)	35 (35.9)	
III	25 (25.0)	21 (21.0)	

laAPR, laparoscopic abdominoperineal resection; taTME, transanal total mesorectal excision; ISR, intersphincteric resection; SD, standard deviation; pCR, pathologic complete response.

### Follow-up results

**Figure 2.**
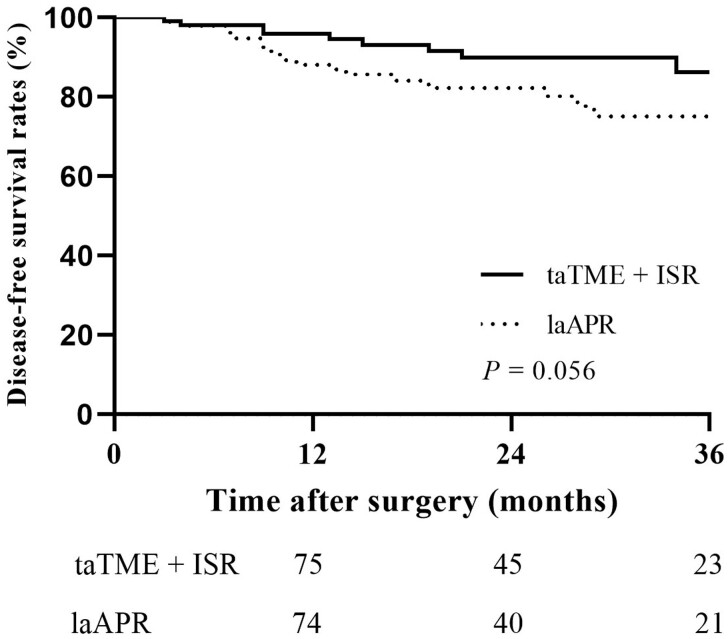
The 3-year DFS rates between laAPR and taTME combined with ISR in patients with low rectal cancer. DFS, disease-free survival; laAPR, laparoscopic abdominoperineal resection; taTME, transanal total mesorectal excision; ISR, intersphincteric resection.

**Figure 3.**
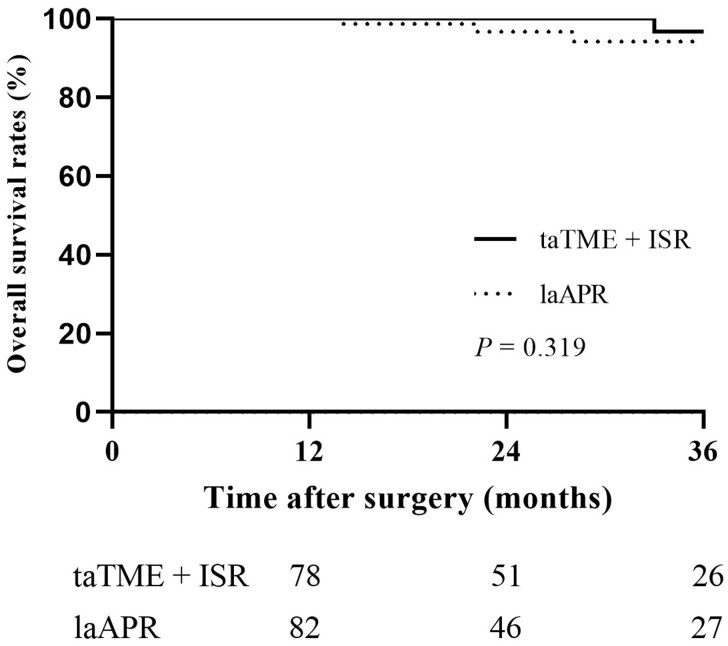
The 3-year OS rates between laAPR and taTME combined with ISR in patients with low rectal cancer. OS, overall survival; laAPR, laparoscopic abdominoperineal resection; taTME, transanal total mesorectal excision; ISR, intersphincteric resection.

**Figure 4.**
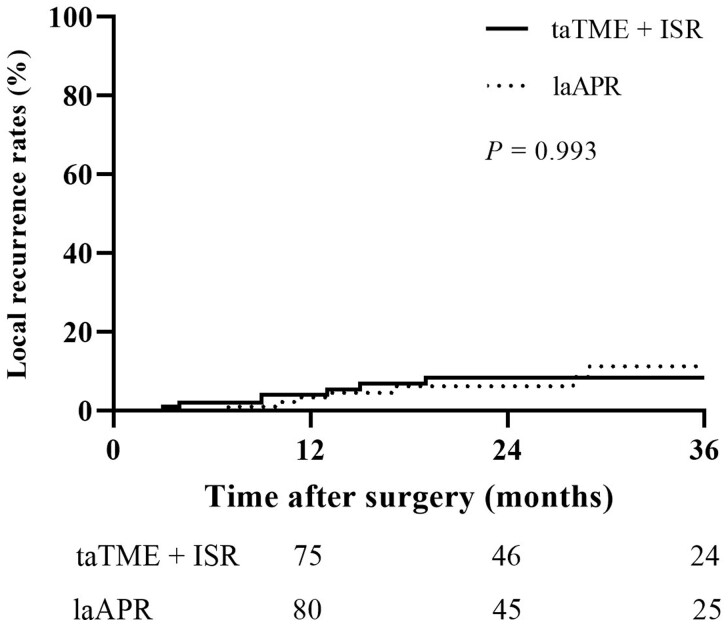
The 3-year LR rates between laAPR and taTME combined with ISR in patients with low rectal cancer. LR, local recurrence; laAPR, laparoscopic abdominoperineal resection; taTME, transanal total mesorectal excision; ISR, intersphincteric resection.

Median follow-up durations were 23 months in both groups. As shown in Figure 2, the 3-year DFS rates were 75.1% in the laAPR group and 86.3% in the taTME-combined-with-ISR group (*P *=* *0.056). Moreover, Figure 3 shows that the 3-year OS rates were 94.2% in the laAPR group and 96.7% in the taTME-combined-with-ISR group (*P *=* *0.319). In addition, within 3 years after surgery, seven patients in each group had LR (Figure 4); it was worth mentioning that most patients were found to have LR within 2 years (five of seven patients in laAPR group and seven of seven patients in taTME-combined-with-ISR group). In the laAPR group, two patients underwent surgery due to LR and one deceased 37 months after surgery; six LR patients received chemotherapy and one deceased without surgery. In the taTME-combined-with-ISR group, three patients with LR received surgery and none deceased; five patients underwent chemotherapy and one died 33 months after surgery.

### LARS score and Wexner score

In the taTME-combined-with-ISR group, all patients had their anal sphincter preserved. Anorectal function for these patients was assessed on the LARS score scale and Wexner incontinence score scale. A total of 86 patients completed these scales, with a median follow-up time of 20 months (range, 1–67 months) after surgeries or stoma closure (if patients received preventive stoma). Fourteen patients were excluded for the following reasons: death due to LR (*n *=* *1), death due to distant recurrence (*n *=* *1), death due to myocardial infarction (*n *=* *1), preventive ileostomy performed during follow-up (*n *=* *5), new ileostomy performed (*n *=* *3), or lost to follow-up (*n *=* *3).

Details on LARS scores are summarized in Table 4. We found that 88.4% of these 86 patients had a bowel movement frequency of one to seven times every day. Nearly half of patients experienced clustering of stools (47.7%) and fecal urgency (50.0%) of stools greater than or equal to once a week. The median LARS questionnaire scores were 27 (range, 0–41), with 39 patients (45.3%) having major LARS. Details on Wexner continence scores are shown in Table 5. The median Wexner continence score was 2 (range, 0–19). A large proportion (70.9%) of patients had satisfactory defecation function following surgery.

**Table 4.** Low anterior resection syndrome (LARS) score

Parameter	taTME + ISR (*n *=* *86)
Total LARS score, median (range)	27 (0–41)
LARS classification, *n* (%)	
No	34 (39.5)
Minor	13 (15.1)
Major	39 (45.3)
Incontinence for flatus, *n* (%)	
Never	40 (46.5)
Less than once a week	24 (27.9)
Equal to or more than once a week	22 (25.6)
Incontinence for liquid stools, *n* (%)	
Never	41 (47.7)
Less than once a week	20 (23.3)
Equal to or more than once a week	25 (29.1)
Bowel frequency per day, *n* (%)	
>7 times	8 (9.3)
4–7 times	29 (33.7)
1–3 times	47 (54.7)
Less than once	2 (2.3)
Clustering of stools, *n* (%)	
Never	32 (37.2)
Less than once a week	13 (15.1)
Equal to or more than once a week	41 (47.7)
Urgency, *n* (%)	
Never	32 (37.2)
Less than once a week	11 (12.8)
Equal to or more than once a week	43 (50.0)

taTME, transanal total mesorectal excision; ISR, intersphincteric resection; SD, standard deviation.

**Table 5.** Wexner incontinence score

Parameter	taTME + ISR (*n *=* *86)
Total Wexner incontinence score, median (range)	2 (0–19)
Anal function, *n* (%)	
Good (≤10)	61 (70.9)
Poor (>10)	25 (29.1)
Anal incontinence for solid stool, *n* (%)	
0	59 (68.6)
1	10 (11.6)
2	12 (14.0)
3	5 (5.8)
4	0
Incontinence for liquid stool, *n* (%)	
0	41 (47.7)
1	3 (3.5)
2	17 (19.8)
3	16 (18.6)
4	9 (10.5)
Incontinence for gas, *n* (%)	
0	41 (47.7)
1	2 (2.3)
2	25 (29.1)
3	15 (17.4)
4	3 (3.5)
Use of pads, *n* (%)	
0	54 (62.8)
1	1 (1.2)
2	6 (7.0)
3	15 (17.4)
4	10 (11.6)
Lifestyle alteration, *n* (%)	
0	44 (51.2)
1	1 (1.2)
2	15 (17.4)
3	17 (19.8)
4	9 (10.5)

taTME, transanal total mesorectal excision; ISR, intersphincteric resection.

## Discussion

Focusing on two highly matched groups with strict inclusion criteria, our study suggested that laAPR involving a larger surgical incision resulted in longer operative times, more intraoperative blood loss, and more post-operative complications compared with taTME combined with ISR. We showed that pathological outcomes and oncologic outcomes including OS, DFS, and LR rates were similar in both groups. Patients receiving taTME combined with ISR had acceptable anorectal function following surgery.

Generally, patients tend to undergo the APR procedure when the tumor is near the anus [18]. In this study, the average height of the tumor measured from the anal verge was 3.2 cm in the laAPR group and 3.3 cm in the taTME-combined-with-ISR group (*P *=* *0.189), and other tumor characteristics were similar after case matching. More surgical procedures resulted in longer operative times and more blood loss in the laAPR group. Large surgical wounds might cause incision infection and fat liquefaction, and thus lead to an extended hospital stay. To preserve the anal sphincter of patients, the taTME-combined-with-ISR group had intestinal anastomosis and reconstruction, which resulted in a anastomotic failure rate of 13.0% at such a low height of tumor in this study, but it was similar to that of 1,594 patients undergoing taTME from the international taTME registry (15.7%) [23]. As for the anastomotic leakage rate, the value of the taTME-combined-with-ISR group (11.0%) was similar to the results from other studies, with a range from 5.3% to 13.9%, utilizing different surgical techniques for low rectal cancer [24–26]. Two patients needed secondary surgery due to anastomotic failure, whereas other patients with anastomotic failure could be treated conservatively.

Previous studies showed that APR had high rates of CRM and was associated with lower survival rates if the surgical resection plane was suboptimal; however, patients receiving APR generally had low tumor height, as well as more locally advanced tumors [27, 28]. A subsequent study including 2,969 patients receiving low anterior resection (LAR) and 1,245 patients undergoing APR considered that the APR group had higher positive rates of CRM than the LAR group (12% vs 8%), but there were no significant difference between the two groups [18]. In our study, it was worth noting that the quality of mesorectal resection specimen, DRM, and CRM in the taTME-combined-with-ISR group showed no significant difference compared with those in the laAPR group. Based on similar post-operative pathological results, this new surgical method can obtain high-quality tumor specimens.

With the same principle of TME, surgeons performed open or laparoscopic surgery for patients with low rectal cancer; different methods had similar oncological outcomes [10, 11]. The introduction of taTME in the past decade has brought clear vision during surgery and made it possible to achieve TME performed precisely in low rectal cancer [12]. Based on the same principle, taTME could bring acceptable oncological outcomes [29, 30]. It was also considered an anus-preserving operation to replace the traditional anal-removal operation, and to avoid permanent colostomy in low or ultra-low rectal cancer. APR was once the gold standard for treating all rectal cancers [3]; as several new techniques to preserve the anal sphincters became available for surgeons [31], the use frequency of APR decreased. The application of new technology will be questioned unless it has sufficient evidence to show its safety. Koyama *et al.* [32] showed that the LR rates after APR occurred in 12.1% of 33 patients, while these were 7.8% in 77 patients in the ISR group. In the same study, the 5-year OS rates were 51.2% in APR, and these were lower than in the ISR group (76.4%). A study with a larger sample size and better match between groups showed that 3-year cumulative LR rates were 3.9% for APR (*n *=* *89) and 7.3% for ISR (*n *=* *89, *P *=* *0.13); the 5-year OS rates were 69.9% for ISR and 67.9% for APR (*P *=* *0.64) [9]. Traditional ISR is not accurate enough to locate the tumor, and it is more difficult to separate the sphincter when patients have obesity or pelvic stenosis. We combined the high-definition magnification and expansion effect of anal endoscopy to make the separation of the sphincter space easier and more precise. Our study suggested that the LR rates were similar between the laAPR group and the taTME-combined-with-ISR group. The 3-year DFS rates (86.3% vs 75.1%, *P *=* *0.056) and 3-year OS rates (96.7% vs 94.2%, *P *=* *0.319) were a bit higher in the taTME-combined-with-ISR group than in the laAPR group, but this difference was not statistically significant. Subsequent long-term follow-up of two groups confirmed the oncological safety and feasibility of taTME combined with ISR.

Surgeons were concerned not only about the oncological results in patients with rectal cancer [10, 11], but also about how to preserve and improve the patients’ anal function. In our research, patients who underwent laAPR had their anus removed and lost the ability to defecate spontaneously. With the improvements in technology and concept, patients who had similar preoperative baselines could achieve anus preservation by receiving taTME combined with ISR surgery. These patients had favorable anorectal function without compromising oncological outcomes. As reported by several studies, patients in the taTME group had similar bowel function for mid and low rectal cancer to those in the laparoscopic TME group [16, 33, 34]. Based on our previous results, the rate of major LARS (45.3%) was comparable to other published studies, ranging from 35% to 82% after taTME surgery, based on different preoperative baselines [14, 16, 33]. The rate of major LARS resulted mainly from high scores of clustering and urgency of stools. However, 29.1% of patients undergoing taTME combined with ISR were classed as having poor anal function, which was lower than that reported in another study in which patients underwent traditional taTME (42%) [14]. This might be explained by the improvement in transanal instruments [35] and surgical techniques [36], both reducing injury to the anus sphincter.

This study had some limitations. On the one hand, this was a retrospective and single-center study. Subsequent prospective multicenter studies were needed to overcome this deficiency. On the other hand, we matched two inclusive groups by 10 preoperative factors to make them similar, but we failed to match for other unknown confounding factors. In addition, a small number of early included cases in the taTME-combined-with-ISR group were performed at the initial stage of the surgeons’ learning curve, while patients in the laAPR group were operated on by surgeons who were experienced. Therefore, this study might not demonstrate all advantages of taTME combined with ISR [36]. Despite several shortfalls, our study directly compared many results of laAPR and taTME combined with ISR to provide surgeons with more options for low rectal cancer surgery.

## Conclusions

Patients who underwent taTME combined with ISR and those undergoing laAPR in patients with low rectal cancer had similar oncological outcomes. Patients undergoing taTME combined with ISR had favorable anorectal function. Further investigation will be needed to confirm our findings.

## Authors’ Contributions

Z.H.L. and Z.W.Z.: conceptualization, methodology, data curation, formal analysis, and writing-original draft. H.Q.J., L.H., S.L.L., W.F.L., and X.W.Z.: data curation, formal analysis, and writing-review and editing. L.K.: conceptualization, project administration, writing-original draft, writing-review and editing, funding acquisition. All authors read and approved the final manuscript.

## Funding

This work was supported by a grant from the Shenzhen “San Ming Projects” Research [Grant No.lc202002 to L.K.]), the Fundamental Research Funds for the Central Universities [Grant No.16ykjc25 to L.K.] and Sun Yat-sen University Clinical Research 5010 Program [Grant No.2016005 to L.K.], and the National Key Clinical Discipline.
